# Previous Catheter Ablation Predicts In-Hospital Restoration of Sinus Rhythm in Patients Presenting with Recent-Onset Atrial Fibrillation—The Retrospective HAMBURG-AF Study

**DOI:** 10.3390/medicina57080776

**Published:** 2021-07-30

**Authors:** Benedict Schulte-Steinberg, Muhammet Ali Aydin, Ana Theresa Moser, Francisco Ojeda, Mahir Karakas

**Affiliations:** 1 Department of Cardiology, Robert-Bosch-Hospital, 70376 Stuttgart, Germany; Benedict.Schulte-Steinberg@rbk.de; 2 Heart Centre Bremen, Kardiologisch-Angiologische Praxis, 28211 Bremen, Germany; dr.a.aydin@icloud.com; 3 Department of Cardiology, University Heart & Vascular Center Hamburg, 20246 Hamburg, Germany; ana_moser@hotmail.de (A.T.M.); F.Ojeda-Echevarria@uke.de (F.O.)

**Keywords:** recent-onset atrial fibrillation, ablation, HAMBURG-AF study

## Abstract

*Background and Objectives:* Atrial fibrillation (AF) is the most common arrythmia of the human heart. Patients mostly present highly symptomatic with dyspnea and tachycardia and have a disproportionate risk of developing heart failure or stroke events. We aimed to evaluate the determinants of early conversion into sinus rhythm during initial stay at the emergency department of a large tertiary care center. *Materials and Methods:* A total of 1384 subjects with recent-onset AF were recruited between October 2014 and April 2017. Patients with longstanding AF were excluded, resulting in a total of 935 patients for the present analysis. *Results:* In multivariate adjusted logistic regression analyses, previous catheter ablation therapy was a strong predictor of conversion in sinus rhythm during the stay in the emergency department, with an odds ratio (OR) of 3.87 (95% CI 2.40, 6.54; *p* < 0.001). In contrast, existing antiarrhythmic medication showed no association with facilitated conversion [OR 0.89 (95%CI 0.65, 1.20); *p* = 0.44]. Likewise, conventional cardiovascular risk factors (hypertension, dyslipidemia, diabetes) were also not associated with conversion during hospital stay. *Conclusion:* This is the first report on the relevance of previous ablation therapy for early restoration of sinus rhythm in recent-onset AF. Although catheter ablation is associated with relevant risk of late recurrence of atrial fibrillation, it seems to have a large benefit for patients with recent-onset AF.

## 1. Introduction

Atrial fibrillation (AF) is a common cardiac disease with an increasing prevalence [1,2,3], pronounced clinical symptoms, a high burden for comorbidities, and an increased mortality [4]. Affected patients have an increased risk for thromboembolic events and the development of heart failure, and catheter ablation has now emerged as key interventional therapy and is recommended for drug-refractory AF, although recurrences of AF are common [5]. It is projected that the number of patients suffering from AF in the European Union (EU) between 2010 and 2060 will double, and the BiomarCaRE consortium could demonstrate in age- and risk factor-adjusted models that incident AF was associated with a 3.5-fold increased risk of death in men and women [6,7,8,9,10,11]. In terms of acute disease, recent-onset AF represents a frequent cause for presentation in the emergency department (ED), and affected patients typically suffer from recent onset of tachycardia, dyspnea, acute anxiety, and a feeling of pressure in their chest. Here, we characterized the real-world patient population presenting with recent-onset AF in the ED, and evaluated the clinical predictors of early restoration of sinus rhythm, since there is no such data available yet.

## 2. Materials and Methods

### 2.1. Patients and Study Variables

**Figure 1:**
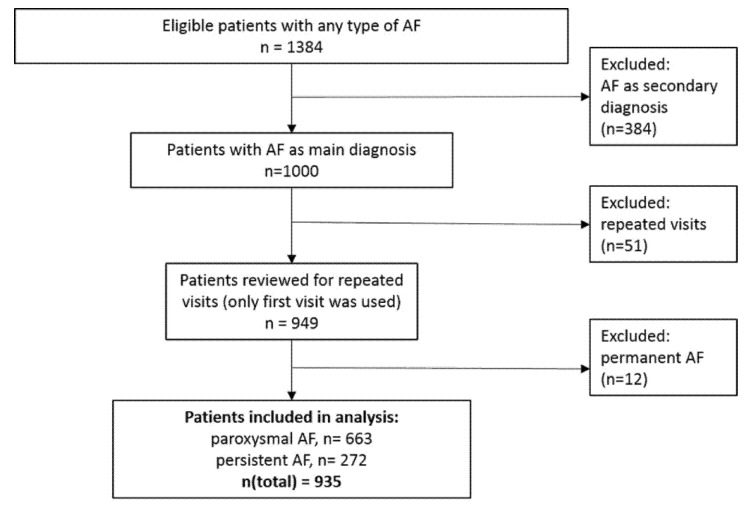
Study flow of HAMBURG-AF.

A total of 1384 subjects were recruited between October 2014 and April 2017. Patients were selected based on their primary admission diagnosis: we included patients with recent-onset AF as a primary diagnosis and excluded patients with AF as secondary diagnosis, as well as patients with long-standing AF. For those with repeated visits we used only the first visit (Figure 1). This resulted in a total of 935 patients for the present analysis.

As shown in Figure 1, a total of 663 patients (71%) of our population were classified as paroxysmal AF. All subjects confirmed participation. Routine clinical variables, as well as characteristics of the EHRA-Score of the European Heart Rhythm Association, were chosen as variables of interest. The study was performed in accordance with the Declaration of Helsinki.

### 2.2. Statistical Analyses

Continuous variables are described using median and 25th and 75th percentiles and categorical variables using absolute and relative frequencies. The Mann–Whitney U test was used for comparisons between groups for continuous variables and the chi-square test for categorical variables. Multivariate age and sex-adjusted binary logistic regression analyses were performed to evaluate potential associations between clinical variables and restoration of sinus rhythm. Afterwards, a single logistic model including the covariates used in the previous models (with the exception of left ventricular ejection fraction because of a large number of missing values) was performed.

## 3. Results

Table 1 shows the patient characteristics, stratified for paroxysmal and persistent AF. The median age was 75 years, and 45.7% were male. Prevalence of coronary artery disease was 21.1%. In 30% of subjects, AF may have been secondary to concomitant bacterial infection.

A total of 44% of those with paroxysmal AF were diagnosed for the first time. In a total of 68.4% of the patients, sinus rhythm was restored during the hospital stay—in 29.6% after administration of medication (potassium/magnesium and/or beta-blocker) and in further 31.6% due to electric cardioversion. The remaining 7.2% converted spontaneously into sinus rhythm. Regarding laboratory findings, patients with persistent AF had a median NT-proBNP level (highest value reported during hospital stay) of 3588 ng/L, while this value was clearly lower in those presenting with paroxysmal AF (2426 ng/L). Approximately one-third (28.3%) of all patients presenting with recent-onset AF were classified as secondary AF patients, of whom the majority (25.1% of the 28.3%) suffered from concomitant acute bacterial infection, such as urinary tract infections or pneumonia. Only about one tenth (9.8%) were present smokers, the clear majority (78.5%) were never-smokers. A total of 64.2% were taking antiarrhythmic medication on a daily basis, of whom 65.8% were under permanent beta-blocker therapy. A total of 27.7% in our population reported previous electric cardioversion, while 18.9% of the patients reported a previous catheter ablation procedure. Regarding restoration of sinus rhythm during actual hospital stay, 66.3% converted within the first 24 h during the hospital stay—22.8% due to administration of medication in ED, and 37.1% due to electric cardioversion during stay in ED.

In further analysis, shown in Table 2, we computed age and sex adjusted logistic regression analysis in order to assess the association between various clinical parameters and the in-hospital restoration of sinus rhythm. The analysis yielded highly significant positive associations for the following variables (listing according to strength of association): previous ablation therapy (*p*-value < 0.001; OR 3.87), previous family history of CAD (*p*-value 0.011, OR 2.57), previous electric cardioversion (*p*-value < 0.001, OR 2.54), substitution therapy with magnesium (*p*-value < 0.001, OR 2.32), administration of beta-blockers (*p*-value < 0.001, OR 1.91). In contrast, statistically significant negative associations with restoration of sinus rhythm in recent-onset AF were found for secondary AF (*p*-value < 0.001, OR 0.37).

## 4. Discussion

Based on a large patient registry, we could identify several predictors of in-hospital restoration of sinus rhythm, with previous catheter ablation therapy as a strong predictor for in-hospital restoration of sinus rhythm in patients presenting with recent-onset AF in the ED. The study thereby confirms the common idea of long-standing benefits of catheter ablation therapy, which has repeatedly been criticized for relatively high recurrence rates of AF of up to 45% within the first year after ablation [12,13,14]. The German ablation registry proved that a large proportion of patients treated by catheter ablation experience a significant symptom reduction due to AF burden reduction, while other groups documented significant reductions in mortality, stroke, and heart failure rates compared with matched general AF populations, and matched population who underwent cardioversion [15,16]. Our report adds to this, by confirming long-term benefits of interventional ablation therapy.

### 4.1. Pathophysiological Insights

One might obviously assume that elimination of AF substrate around the pulmonary veins may have contributed to sinus restoration. Despite this assumption, recently, Nielsen et al. published a genome-wide association-study (GWAS) testing almost nine million genetic variants in more than 36,000 AF-individuals, identifying seven risk loci [17]. Similarly, Husser and coworkers assessed rhythm outcome of catheter ablation of AF in a gene-based analysis of GWAS data [18]. This study exposed two genes (ITGA9, SOX5), which were significantly associated with left atrial low voltage areas and left atrial diameter and subsequently with AF recurrence after catheter ablation therapy [18]. Extending this finding, others found rhythm control after ablation therapy to also be determined by atrial remodeling and circulating biomarkers like transforming growth factor beta-1 [19], indicating that beyond non-modifiable genetic determination, a certain modulation of rhythm control and response is existing. Moreover, as one of the most innervated organs, the electrophysiological activity of the human heart is significantly modulated by extrinsic and intrinsic nerve fibers [20]. The sympathetic and parasympathetic nervous system can influence ion channels, ion currents and atrial electrophysiology and thereby facilitate electrical triggers and reentry mechanisms [20]. By stimulating beta-adrenergic receptors, the activity of hyperpolarization-activated inward current (If) can be intensified, whereas stimulation of atrial alpha-adrenergic receptors can reduce inwardly rectifying Kir current (IK1) activity [20]. By stimulation of alpha1- and beta1-adrenoceptors, increasing activity in the rat pulmonary vein could be demonstrated [21,22]. Altogether, this illustrates the possibility for modulation and underlines the suggestion that catheter ablation might modulate electrophysiological automaticity, thereby facilitating early restoration of sinus rhythm in the mid- and long-term.

Of note, a family history of CAD also showed a significant association. Importantly, it was only reported in 7.2% of the patients, so this might be a simple finding by chance. On the other hand, there is a known coincidence between CAD and AF. It might be that patients with known CAD have experienced AF earlier in life, and therefore AF is much more obstinate in these patients.

### 4.2. Translational Perspective

The results provide insight into determinants of in-hospital conversion into sinus rhythm. They confirm the common idea of long-standing benefits of catheter ablation therapy that has repeatedly been criticized for common recurrences in recent years.

### 4.3. Strengths and Limitations

Our data represents real-world evidence from Germany, and thereby might be restricted regarding applicability to other, especially non-Western, regions. Importantly, due to missing follow-up beyond discharge, our findings do not allow conclusions regarding midterm outcomes, since duration of sinus rhythm after restoration was not assessed. On the other hand, diagnoses were made by highly skilled physicians working in the ED and were audited subsequently by two independent physicians.

## 5. Conclusions and Limitations

Within the HAMBURG-AF study, we created, to the best of our knowledge, the first retrospective real-world report on the determinants of in-hospital restoration of sinus rhythm in subjects presenting with recent-onset AF in the ED. The study documents the long-standing benefits of interventional catheter ablation therapy: within age- and sex-adjusted logistic regression analysis, previous interventional catheter ablation therapy was a solid predictor of early conversion into sinus rhythm during hospital stay. These results further underline the importance of catheter ablation therapy in AF, and clearly relativize the observed common recurrences. An important limitation is that we have defined successful hospital sinus rhythm restoration within a 24 h window as the endpoint, and not a longer period. It might be that this created a bias, as in patients with previous cardioversion or ablation, it is usually easier to define what they will react to, and therefore it is easier to intervene and obtain success in a 24 h window. Moreover, the data on LVEF were missing in many patients and therefore were excluded from presentation and analyses.

## Figures and Tables

**Table 1:** Patient characteristics.

	All (*N* = 935)	Paroxysmal (*N* = 663)	Persistent (*N* = 272)	*p*-Value
Male No. (%)	437 (46.7)	303 (45.7)	134 (49.3)	0.36
Age (years)	75.0 (65.0, 81.0)	75.0 (65.0, 81.0)	74.0 (65.0, 79.0)	0.064
First diagnosis of AF at ED No. (%)	342 (36.7)	294 (44.5)	48 (17.7)	<0.001
Hypertension No. (%)	644 (69.0)	456 (69.0)	188 (69.1)	1.00
Dyslipidemia No. (%)	147 (16.8)	90 (14.7)	57 (21.6)	0.016
Diabetes mellitus No. (%)	125 (13.4)	86 (13.0)	39 (14.3)	0.65
Family history of CAD No. (%)	67 (7.2)	34 (5.2)	33 (12.2)	<0.001
CAD No. (%)	200 (21.5)	139 (21.1)	61 (22.4)	0.72
max NT-proBNP (ng/L)	2779 (1081, 6262)	2426 (1015, 5719)	3588 (1549, 6951)	0.020
Concomitant bacterial infection No. (%)	265 (28.3)	204 (30.8)	61 (22.4)	0.013
Current smoker No. (%)	91 (9.8)	67 (10.1)	24 (8.8)	0.027
Never smoked No. (%)	732 (78.5)	528 (79.9)	204 (75.0)	
Ex-smoker No. (%)	110 (11.8)	66 (10.0)	44 (16.2)	
Conversion during hospital stay No. (%)	610 (66.3)	443 (68.4)	167 (61.4)	0.050
Conversion due to medication No. (%)	210 (22.8)	193 (29.6)	17 (6.3)	<0.001
Conversion due to electric CV No. (%)	345 (37.1)	208 (31.6)	137 (50.4)	<0.001
Secondary AF No. (%)	234 (25.1)	191 (28.9)	43 (15.8)	<0.001
Previous ablations No. (%)	176 (18.9)	75 (11.3)	101 (37.4)	<0.001
Existing antiarrhythmic medics No. (%)	598 (64.2)	400 (60.6)	198 (72.8)	<0.001
Administration of K+ No. (%)	319 (34.2)	228 (34.5)	91 (33.5)	0.82
Administration of Mg+ No. (%)	613 (65.6)	433 (65.4)	180 (66.2)	0.88
Administration of beta-blocker No. (%)	615 (65.8)	438 (66.2)	177 (65.1)	0.81
Hx previous electric cardioversion No. (%)	258 (27.7)	108 (16.3)	150 (55.6)	<0.001

AF = atrial fibrillation; ED = emergency department; CAD = coronary artery disease; NT-proBNP = N-terminal pro b-type natriuretic peptide; K+ = potassium; Mg+ = magnesium; Hx = history of. For continuous variables median (25th percentile, 75 percentile) is given. For binary variables absolute and relative frequencies are shown.

**Table 2:** Summary of age and sex adjusted logistic regressions for restoration of sinus rhythm during hospital stay.

	OR (95% CI)	*p*-Value
Secondary AF	0.37 (0.26, 0.51)	<0.001
Previous ablations	3.87 (2.40, 6.54)	<0.001
Existing antiarrhythmic medication	0.89 (0.65, 1.20)	0.44
Administration of K+	1.35 (0.99, 1.85)	0.056
Administration of Mg+	2.32 (1.72, 3.13)	<0.001
Administration of beta-blocker	1.91 (1.41, 2.57)	<0.001
First diagnosis of AF at ED presentation	0.87 (0.65, 1.17)	0.36
Hypertension	1.11 (0.80, 1.52)	0.53
Dyslipidemia	1.12 (0.75, 1.67)	0.59
Diabetes mellitus	0.69 (0.46, 1.04)	0.075
Family history of CAD	2.57 (1.30, 5.70)	0.011
Known CAD	0.87 (0.62, 1.24)	0.43
Previous electric cardioversion	2.54 (1.77, 3.70)	<0.001

AF = atrial fibrillation; K+ = potassium; Mg+ = magnesium; ED = emergency department; CAD = coronary artery disease.

## Data Availability

Data supporting reported results can be obtained by the authors.
